# A Witches’-Broom Disease of Cultivated Strawberry Associated with ‘*Candidatus* Phytoplasma Rubi’-Related Strains in Southern Italy

**DOI:** 10.3390/plants14182914

**Published:** 2025-09-19

**Authors:** Carmine Marcone, Carmine Palmieri, Alberto Sellitto

**Affiliations:** Department of Pharmacy, University of Salerno, I-84084 Fisciano, Salerno, Italy; c.palmieri20@studenti.unisa.it (C.P.); a.sellitto19@studenti.unisa.it (A.S.)

**Keywords:** *Fragaria* × *ananassa*, crown proliferation symptoms, etiology, PCR assays, 16SrV-E subgroup, rubus stunt, phylogenetic analysis, rDNA, *groEL* gene

## Abstract

In the Campania region of southern Italy, a formerly undescribed witches’-broom disease of cultivated strawberry characterized by symptoms similar to those of strawberry witches’-broom and multiplier diseases occurring in North America, has been observed. Strawberry witches’-broom and multiplier diseases are not known to occur in Europe. To elucidate the etiology of the new strawberry disease occurring in southern Italy and to determine the taxonomic position of the presumable causal agent, field observations and PCR assays using universal and group-specific phytoplasma primers followed by multigene sequence analysis were carried out. All of the symptomatic strawberry plants examined tested phytoplasma positive with universal primers and primers specific to the elm yellows (EY) phytoplasma group or 16SrV group. The percentage of diseased plants in the fields was about 30%. Data obtained from sequence and phylogenetic and virtual RFLP analyses of PCR-amplified rDNA (16S rDNA and 16S/23S rDNA spacer region), *rpsV* (*rpl22*) and *rpsC* (*rps3*), *map*, *imp* and *groEL* gene sequences, showed that the diseased strawberry plants harbored phytoplasma strains which were identical or nearly identical to each other and to strains of the rubus stunt (RuS) agent ‘*Ca*. Phytoplasma rubi’, a member of the 16SrV group, subgroup 16SrV-E. The 16S rDNA sequence similarity among the strawberry-infecting phytoplasma strains ranged from 99.1 to 99.9%. These strains shared the same range of 16S rDNA sequence similarity with RuS phytoplasma strains including the reference strain RUS of ‘*Ca*. Phytoplasma rubi’. This is the first report on the occurrence of RuS phytoplasma in naturally affected strawberry plants.

## 1. Introduction

Several phytoplasma diseases have been reported to affect the cultivated strawberry (*Fragaria* × *ananassa* Duch.) wherever this high-value crop is grown. These diseases include strawberry aster yellows, green petal, lethal yellows, lethal decline, fruit phyllody, witches’-broom, multiplier, yellows, red leaf and others [1,2,3,4,5,6,7,8]. Phytoplasmas are obligate, phloem-restricted prokaryotic pathogens of the *Mollicutes* class associated with diseases of a large number of plant species worldwide [9]. These pathogens are disseminated by phloem-feeding homopteran insects, mainly leafhoppers and planthoppers, and less frequently psyllids, which transmit them in a persistent, propagative manner [10]. Phytoplasmas are not mechanically transmissible. However, they can be transmitted by grafting and dodder (*Cuscuta* spp.) [11,12]. Many phytoplasmas have been transmitted from naturally infected plants to experimental hosts including periwinkle (*Catharanthus roseus*) via dodder bridges. Phytoplasmas cause a wide range of symptoms that are either specific such as virescence, phyllody and witches’-broom, or non-specific such as yellowing, stunting and decline [13]. These as-yet uncultured prokaryotes are currently classified within the provisional genus ‘*Candidatus* Phytoplasma’ based primarily on 16S rDNA sequence analysis [14]. Within this genus, 37 distinct taxonomic groups (or 16Sr groups), more than 150 subgroups and 56 ‘*Candidatus* Phytoplasma’ species have been delineated [14,15,16,17,18]. Also, multi-locus sequence analysis of various housekeeping genes is widely used as an additional tool for characterization and differentiation of phytoplasmas and delineation of new taxonomic entities with the ‘*Candidatus* Phytoplasma’ genus [15,16].

Phytoplasma strawberry diseases differ in their geographic distribution, economic importance, symptoms on natural and experimental hosts, insect vectors and number and size of the taxonomic groups and subgroups of the associated phytoplasmas. Strawberry aster yellows disease has been described in North America [1,19]. This disease is mainly characterized by flower and fruit abnormalities, which are rare and/or never pronounced in other phytoplasma diseases affecting strawberry. Phytoplasmas of the aster yellows (AY) group or 16SrI group, subgroups 16SrI-A and 16SrI-B, are associated with the disease [19]. Strawberry green petal disease has been reported in North America, Mexico, Argentina, Chile, Australia and Europe [2,20]. The most characteristic symptom from which the name of the disease is derived is the presence of flowers with green petals. This disease is associated with phytoplasmas belonging to subgroups 16SrI-C, 16SrI-R, 16SrVII-C, 16SrXII-B, 16SrXIII-A, 16SrXIII-B, 16SrXIII-F, 16SrXIII-I and 16SrXIII-K [21,22,23,24,25,26,27,28,29,30,31,32]. Strawberry lethal yellows disease is known to occur in Australia and New Zealand [33,34]. The main symptoms include purple coloration of older leaves, yellowing of younger leaves and death of affected plants. Strawberry lethal yellows disease is associated with phytoplasmas of subgroups 16SrII-D and 16SrXII-B [25,35,36,37]. Strawberry lethal decline was first observed in the north-western United States and was later reported in British Columbia [38]. The most characteristic symptom of this disease is upward rolling of leaves, which are dwarfed, chlorotic, somewhat bronzed on the upper surface, reddish or purplish on the lower surface and have unequal-sized leaflets. The phytoplasma etiology of strawberry lethal decline is based on electron microscope observations. However, identity and taxonomic position of the associated phytoplasma are unknown. Strawberry fruit phyllody has been observed in Maryland, West Virginia and Brazil. The most obvious symptom is the appearance of phylloid fruits. The disease is associated with phytoplasmas of subgroups 16SrI-K, 16SrI-R, 16SrIII-B, 16SrIII-K, 16SrVI-B and 16SrXIII-J [39,40,41,42,43]. Strawberry witches’-broom and multiplier diseases have been reported in North America [3,44,45]. Both diseases induce pronounced and characteristic symptoms in affected strawberry plants which are very similar. These symptoms include crown proliferation, small leaflets borne on short, spindly petioles and shortened runners. According to published descriptions, witches’-broom seems to cause a more brooming effect than that of multiplier disease [46]. Also, the slight differences in symptomatology between the two diseases that are reported in the literature could be ascribed to different environmental conditions and/or to different susceptibility of the various cultivars examined [3]. Some authors regarded them as identical diseases (for references see [47]). Strawberry witches’-broom and multiplier diseases have been found to be associated with 16SrI-K, 16SrIII-B, 16SrVI-B and 16SrVI-C subgroup phytoplasmas [23,39,48,49]. Among these, subgroup 16SrVI-B phytoplasma, termed *Fragaria multicipita* phytoplasma, was reported to be the causal agent of the aberrant growth form of *Fragaria virginiana*. This aberrant growth form, which is typical of the strawberry multiplier disease, was erroneously considered as a new species of strawberry, *Fragaria multicipita* [48,49]; therefore, the rare plant species *F. multicipita* is an invalid taxon [48,49]. Strawberry yellows disease has been reported in Lithuania. Symptoms shown by affected plants were foliar yellowing and general stunting. The disease is associated with a 16SrXII-E subgroup phytoplasma (=‘*Ca*. Phytoplasma fragariae’) [6]. Strawberry red leaf disease, also termed strawberry redness disease, has been found in Argentina. The main symptom of this disease, which is lethal, is a red discoloration of the upper surface of mature leaves. Strawberry red leaf disease is associated with phytoplasmas of subgroups 16SrXIII-F and 16SrIII-J and of three newly identified 16SrXIII subgroups which have not yet been officially named [7,50]. There are several other reports on the occurrence of phytoplasma infections in diseased strawberry plants with symptoms similar and/or identical to those of the above-mentioned diseases. In California and Maryland, strawberry plants showing symptoms of yellowing and stunting were infected by 16SrIII-B and 16SrVI-A subgroup phytoplasmas [39,51], whereas in Cuba, strawberry plants with symptoms of leaf yellowing, leaf reddening and stunting proved to be infected by a 16SrI-F subgroup phytoplasma [52]. In northern Italy, yellows-affected strawberry plants with typical symptoms of the marginal chlorosis disease [5], i.e., purple discoloration and chlorotic margin of leaves, small and cupped leaves, shortened petioles, stunting, floral abnormalities, reduced root growth and plant death, were found to host 16SrXII-A subgroup phytoplasma [53]. This pathogen is also known to occur singly and/or in association with the walled bacterium ‘*Ca*. Phlomobacter fragariae’ in marginal chlorosis-affected strawberry plants in France [54,55,56]. Phytoplasmas of groups 16SrI and 16SrXII were also identified in strawberry plants showing symptoms of yellowing, stunting, reduced leaf size and virescence in Spain [57].

In southern Italy, notably in Campania and Basilicata regions, strawberry is e widely grown as fresh and shipping market fruit crop. Planting time is generally in October with plants raised in nurseries located mainly in foreign countries such as Poland and Spain. Following the end of production season in June of the following year, the strawberry plants are removed and the beds are subjected to solarization in order to be replanted with the same crop in October. During spring 2024, strawberry plants showing symptoms suggestive of phytoplasma infection were observed in three neighboring commercial fields located near Caserta in the Campania region, a major strawberry-growing area. The symptoms were similar to those described for witches’-broom and multiplier diseases of strawberry. As mentioned above, these diseases are not known to occur in Europe. Therefore, the work presented here was carried out to elucidate the etiology of the new strawberry disease occurring in southern Italy and to determine the taxonomic position of the presumable causal agent. This was accomplished by employing field observations and PCR assays followed by multigene sequence analysis. The results indicated that phytoplasma strains identical or nearly identical to those of the rubus stunt (RuS) agent ‘*Ca*. Phytoplasma rubi’, which were previously not known to occur in naturally affected strawberry plants, were associated with the disease investigated. The strawberry-infecting phytoplasma strains occurring in southern Italy are hereafter referred to as strawberry witches’-broom (StraWB) phytoplasma strains.

## 2. Results

### 2.1. Field Observations

**Figure 1:**
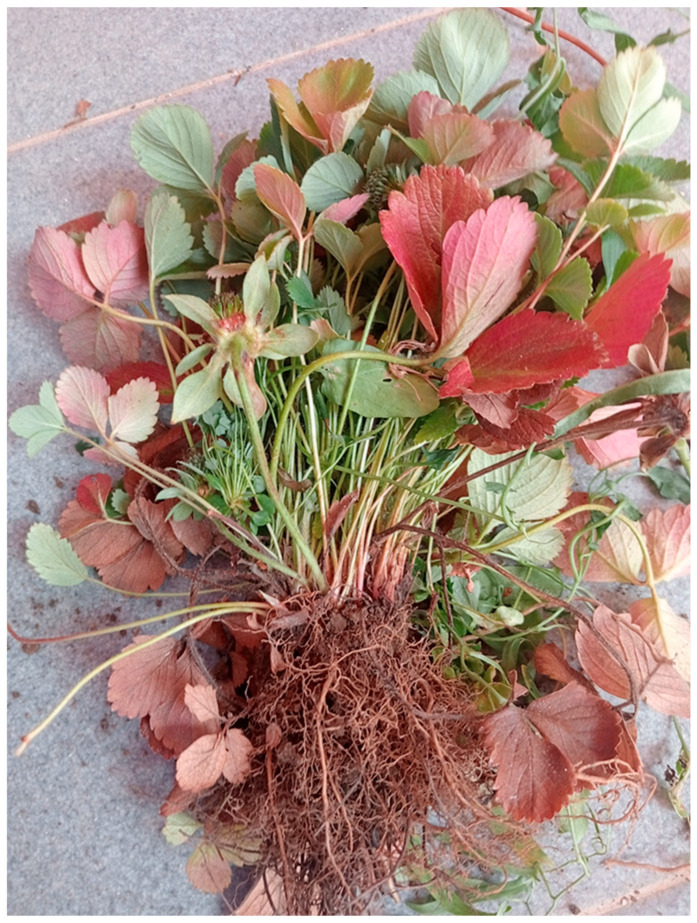
Strawberry witches’-broom (StraWB)-affected cultivated strawberry (*Fragaria* × *ananassa*) plant showing mainly stunting and excessive development of erect, stiff and spindly petioles bearing small leaves, which gave the plant a bushy or brooming appearance. The external leaves are red and cup-shaped.

The symptoms of diseased strawberry plants strikingly resembled those described for strawberry witches’-broom and multiplier diseases [46]. The most conspicuous symptoms were stunting and crown proliferation. Affected plants were dwarfed and displayed multiple-branch crowns with excessive numbers of erect, stiff and spindly petioles bearing small leaves, which confer the plants a bushy or brooming appearance (Figure 1).

**Figure 2:**
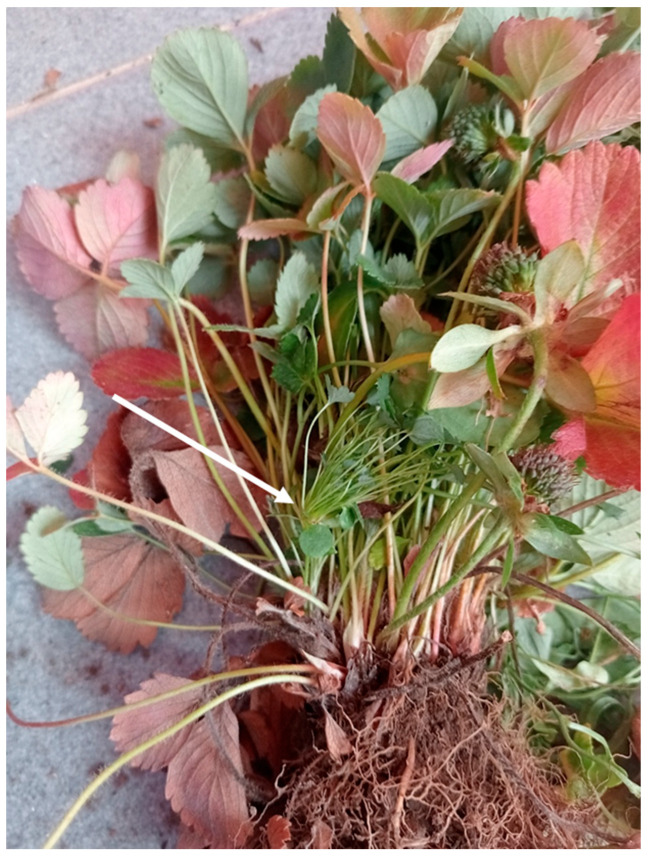
Diseased cultivated strawberry plant showing crown proliferation with a cluster (arrow) of adventitious thin petioles arising from the apical end of a branch crown. Altered berries, elongated sepals, foliar reddening and cupping of leaves are also present.

Clusters of proliferating thin petioles arising from the terminal growing point of a branch crown, instead of flower clusters, were noticeable (Figure 2).

**Figure 3:**
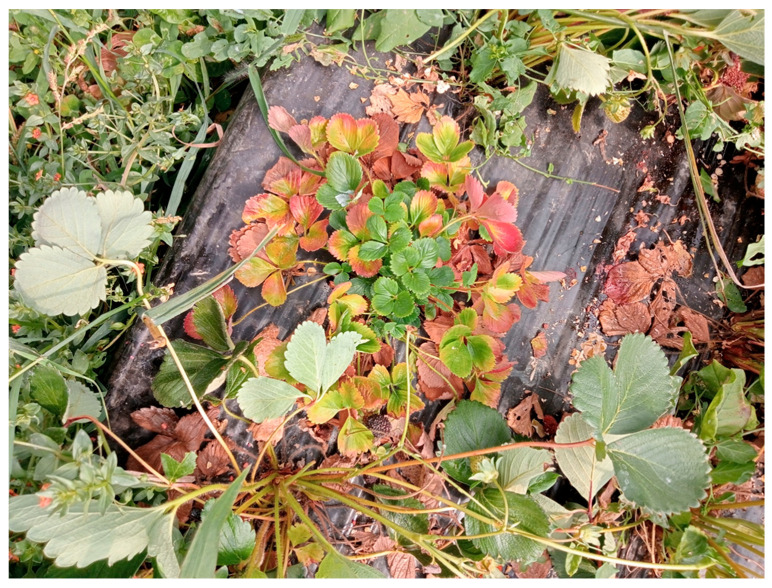
Symptoms of stunting, small leaves, cupped leaves, yellowing, reddening and witches’-broom on a strawberry witches’-broom (StraWB)-affected strawberry plant. On the bottom, a healthy-looking strawberry plant.

Runner production was greatly impaired in diseased plants. However, whenever formed, runners were shortened and daughter plants became established close to mother plants, thereby enhancing the bushy appearance of the growth habit of diseased plants. Young leaves showed yellowing symptoms and were generally cupped upwardly, whereas older leaves often reddened, laid flat on the ground, and soon turned brown (Figure 3).

Flower clusters (stalks) of affected plants were unfruitful and considerably reduced in number. Virescent petals on partly or fully sterile flowers, virescent petals with phyllody of achenes, entirely phylloid flowers and elongated sepals were observed in some instances (Figures S1 and S2). Roots of affected plants showed a reduced growth in comparison to healthy strawberry plants. The symptoms of affected plants became increasingly severe over the observation period. Diseased strawberry plants had a random distribution in the fields. Diseased plants sometimes occurred in groups scattered over several rows. In other instances, several adjacent diseased plants in the border rows of the plastic tunnels were observed. The percentage of diseased plants was about 30% in each field investigated.

### 2.2. Phytoplasma Detection and Identification

All symptomatic strawberry plants examined (60 plants) tested phytoplasma positive in PCR assays using the universal phytoplasma primer pairs P1/P7, P1A/P7A and R16F2n/R2 and the group-specific primer pair fB1/rULWS, all directed to rDNA sequences. However, in samples from 12 symptomatic strawberry plants examined, the target DNA could be amplified only through nested PCR assays in which the initial PCR round carried out with the primer pair P1/P7 was followed by a second round using either P1A/P7A and R16F2n/R2 or fB1/rULWS. Also, the target DNA was amplified with universal primer pairs from all previously characterized phytoplasma strains used for comparison and from strains RuS, EY, ALY, SpaWB and FD using the primer pair fB1/rULWS. No amplification products were obtained from diseased strawberry plants with group-specific primer pairs fStol/rStol, fAY/rAY and R16(III)F2/R1 whereas amplification products of the expected size were obtained with these primers from phytoplasma strains used for comparison of the corresponding groups. Neither by direct nor by nested PCR was DNA amplified from non-symptomatic strawberry plants and healthy controls. Non-ribosomal DNA (rDNA) primer pairs yielded amplification products of the expected size from all symptomatic strawberry plants. The primer pair Fra4/Fra5, specific for ‘*Ca*. Phlomobacter fragariae’, did not amplify the target DNA in any of the strawberry samples tested.

**Figure 4:**
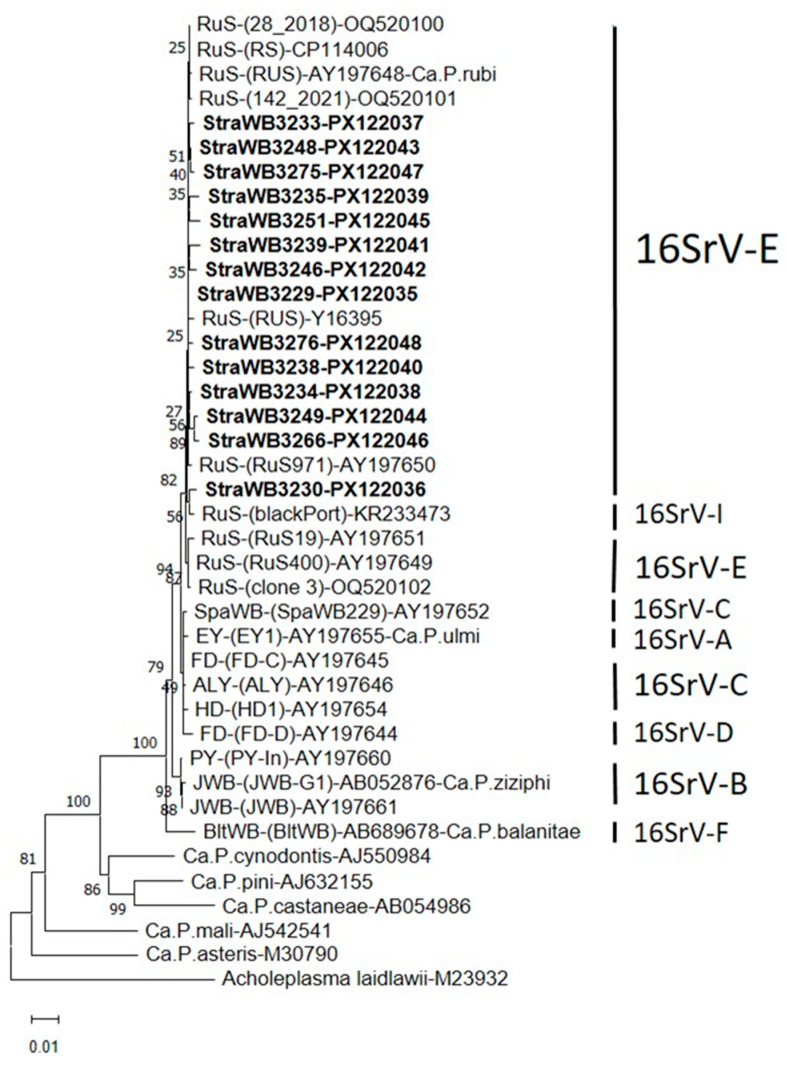
Phylogenetic tree constructed using the neighbor-joining method [58] with 16S rDNA sequences from strawberry witches’-broom (StraWB) phytoplasma strains detected in southern Italy (in bold type), rubus stunt (RuS) phytoplasma strains, 16SrV-A, 16SrV-B, 16SrV-C, 16SrV-D, and 16SrV-F subgroup phytoplasmas and a number of formally described ‘*Candidatus* Phytoplasma’ species. *Acholeplasma laidlawii* was used as the outgroup. The bar represents a phylogenetic distance of 0.01 nucleotide substitutions per site. The GenBank accession number is provided for each phytoplasma. Bootstrap values are shown on branches of the phylogenetic tree.

Nucleotide sequence comparisons of 16S rDNA sequences revealed that the detected StraWB phytoplasma strains were closely related to each other and to strains of the rubus stunt (RuS) agent ‘*Ca*. Phytoplasma rubi’, available in the GenBank database, which all originated from RuS-affected *Rubus* plants in Europe (Table S1). The 16S rDNA sequence similarity among the StraWB phytoplasma strains ranged from 99.1 to 99.9%. These strains shared the same range of 16S rDNA sequence similarity with RuS phytoplasma strains including the reference strain RUS of ‘*Ca*. Phytoplasma rubi’. Comparisons of StraWB phytoplasma strains with other members of the 16SrV phytoplasma group showed the highest 16S rDNA sequence similarity values of 99.2–99.7% with ALY and FD-C strains of the 16Sr-C subgroup. In phylogenetic analysis of 16S rDNA sequences, StraWB phytoplasma strains clustered tightly together with RuS phytoplasma strains. Flanked to the cluster of StraWB and RuS phytoplasma strains were phytoplasmas of 16SrV-A (EY1), 16Sr-C (SpaWB229, FD-C, ALY and HD1) and 16SrV-D (FD-D) subgroups (Figure 4).

**Figure 5:**
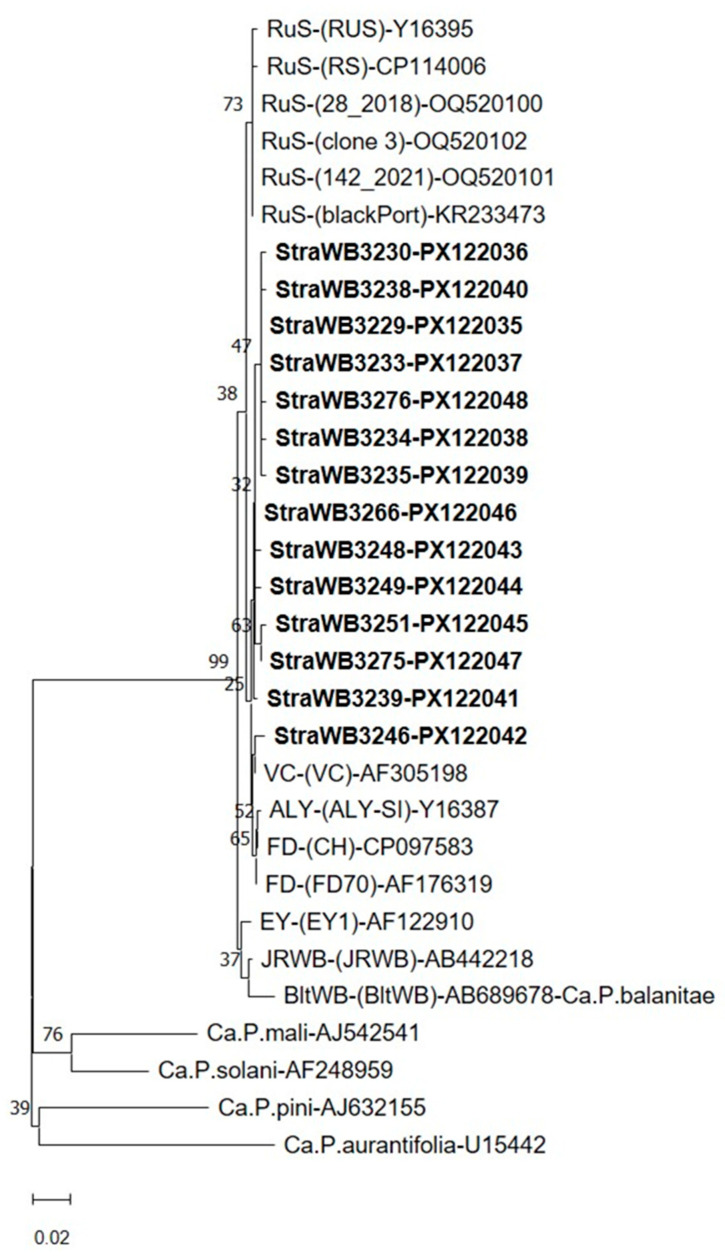
Phylogenetic tree constructed using the neighbor-joining method [58] with 16S/23S rDNA spacer region sequences from strawberry witches’-broom (StraWB) phytoplasma strains detected in southern Italy (in bold type), rubus tunt (RuS), Virginia creeper (VC), alder yellows (ALY), Flavescence dorée (FD), elm yellows (EY), Japanese raisin witches’-broom (JRWB) and Balanites witches’-broom (BltWB) phytoplasma strains, and a number of formally described ‘*Candidatus* Phytoplasma’ species. ‘Ca. Phytoplasma aurantifolia’ was used as the outgroup. The bar represents a phylogenetic distance of 0.02 nucleotide substitutions per site. The GenBank accession number is provided for each phytoplasma. Bootstrap values are shown on branches of the phylogenetic tree.

StraWB phytoplasma strains shared 98.3–100% sequence similarity in the 16S/23S rDNA spacer region. The sequence similarity between StraWB phytoplasma strains and RuS phytoplasma strains (three strains from the Czech Republic and one strain each from Italy, Germany and Portugal) (Table S1) in the 16S/23S rDNA spacer region ranged from 98.3 to 99.3%. Of the other phytoplasmas of the 16SrV group, the most closely related to StraWB phytoplasma strains at 16S/23S rDNA level were strains FD (FD70 and CH of 16Sr-D subgroup) which shared with them sequence similarity values of 98.8–99.6%. StraWB phytoplasma strains clustered tightly together with RuS phytoplasma strains in the phylogenetic analysis of 16S/23S rDNA spacer region sequences and were clearly distinguished from other 16SrV group phytoplasmas (Figure 5).

**Figure 6:**
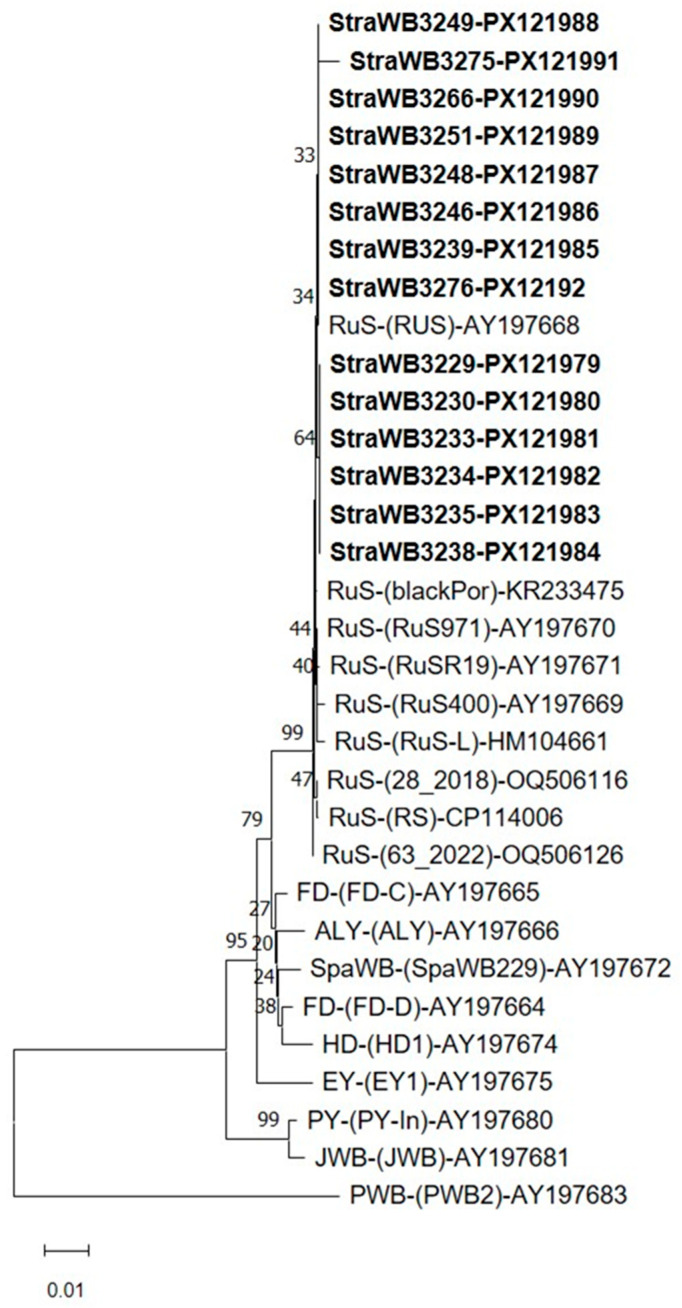
Phylogenetic tree constructed by the neighbor-joining method [58] with ribosomal protein (rp) *rpsV* (*rpl22*) and *rpsC* (*rps3*) gene sequences, from strawberry witches’-broom (StraWB) phytoplasma strains (in bold type), rubus stunt (RUS), Flavescence dorée (FD), alder yellows (ALY), spartium witches’-broom (SpaWB), hemp dogbane yellows (HD), elm yellows (EY), peach yellows (PY) and jujube witches’-broom (JWB) phytoplasma strains. Potato witches’-broom (PWB) phytoplasma strain PWB2 was used as the outgroup. Bar represents a phylogenetic distance of 0.01 nucleotide substitutions per site. GenBank accession number is provided for each phytoplasma. Bootstrap values are shown on branches of the phylogenetic tree.

Sequence similarity of ribosomal protein (rp)-encoding genes *rpsV* (*rpl22*) and *rpsC* (*rps3*) varied from 99.4 to 100% among StraWB phytoplasma strains and was 99.2–100% between StraWB and RuS phytoplasma strains. Sequence similarity of rp genes between StraWB phytoplasma strains and other 16SrV subgroup members was lower, being 98.4–98.9%, 98.2–98.7%, 98.1–98.6%, 98.0–98.5%, 97.1–98.3%, 97.0–97.5%, 96.1–96.6% and 95.9–96.4% for FD-C, FD-D, SpaWB229, ALY, HD1, EY1, PY-In and JWB strains, respectively. StraWB phytoplasma strains showed in the *rpsV* (*rpl22*) and *rpsC* (*rps3*) genes the oligonucleotides 5′-TTATTAAAAAGCGCTGTTGCAA-3′, 5′-TATTTGTCGACGAAGGTTTGCG-3′ and 5′-TAGTTTAATCAATAAAATAGAA-3′ which are specific to ‘*Ca*. Phytoplasma rubi’ [59]. In phylogenetic analysis of rp gene sequences, StraWB phytoplasma strains clustered tightly together with RuS phytoplasma strains. Flanked to the cluster of StraWB and RuS phytoplasma strains were 16SrV-C (FD-C, ALY, SpaWB229 and HD1) and 16SrV-D (FD-D) subgroup phytoplasmas (Figure 6).

**Figure 7:**
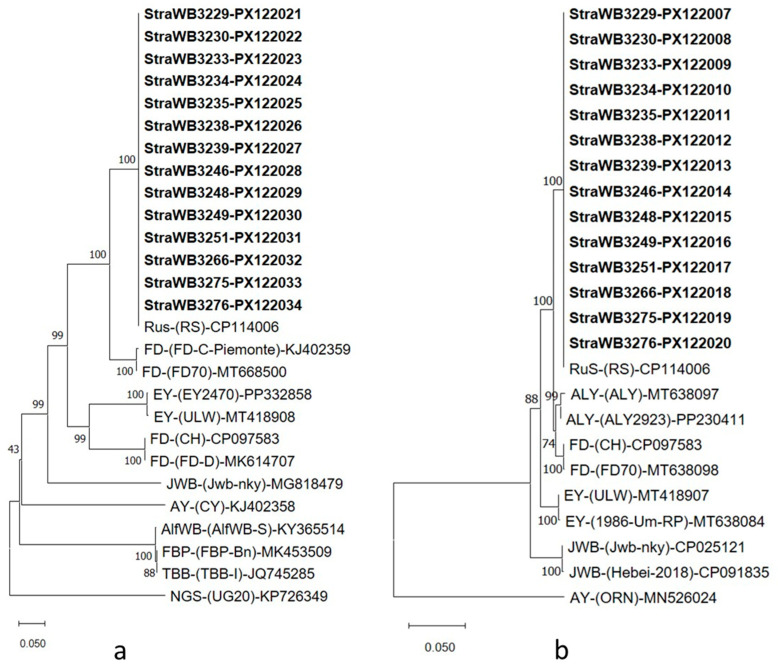
Phylogenetic trees constructed by the neighbor-joining method [58] with *imp* (**a**) and *groEL* (**b**) gene sequences from strawberry witches’-broom (StraWB) phytoplasma strains (in bold type), rubus stunt (RuS) and other 16SrV group phytoplasma strains, and a number of reference phytoplasmas. Napier grass stunt (NGS) phytoplasma strain UG20 (**a**), and aster yellows phytoplasma strain ORN (**b**) were used as the outgroups. The bar represents a phylogenetic distance of 0.05 nucleotide substitutions per site. The GenBank accession number is provided for each phytoplasma. Bootstrap values are shown on branches of the phylogenetic trees.

StraWB phytoplasma strains showed identical *map* gene sequences, which encompass the ‘*Ca*. Phytoplasma rubi’-specific oligonucleotides 5′-AACATAAAGGTTATTTTGTAGAT-3′ and 5′-CATGGTATTGGAAAAAAATTACA-3′ [59], and proved to be indistinguishable from all RuS phytoplasma strains compared. StraWB phytoplasma strains shared 98.2%, 98.1%, 98.1%, 98.1%, 97.9%, 97.8%, 97.6%, 97.5%, 97.3% and 96.9% *map* sequence similarity with HD1, PGY-A, PGY-B, FD (FD70), ALY, FD (VI04-C28), PGY-C, SpaWB (SI04-S4), EY (E04-D714) and FD (V00-SP5) strains, respectively. The phylogenetic relationships of StraWB phytoplasma strains at *map* gene sequence level to each other and to other 16SrV group phytoplasmas are shown in Figure S3. Also, StraWB phytoplasma strains were identical to each other and to German RuS strain RS in each *imp* and *groEL* gene. Of the other 16SrV group phytoplasmas, the most closely related to StraWB phytoplasma strains were FD (FD70) and ALY (ALY2923) strains which shared with them 89.6% *imp* and 98.5% *groEL* sequence similarity, respectively. The phylogenetic relationships of StraWB phytoplasma strains based on *imp* and *groEL* gene sequences to each other and to the most closely related phytoplasmas are shown in Figure 7.

### 2.3. Virtual RFLP Analysis

**Figure 8:**
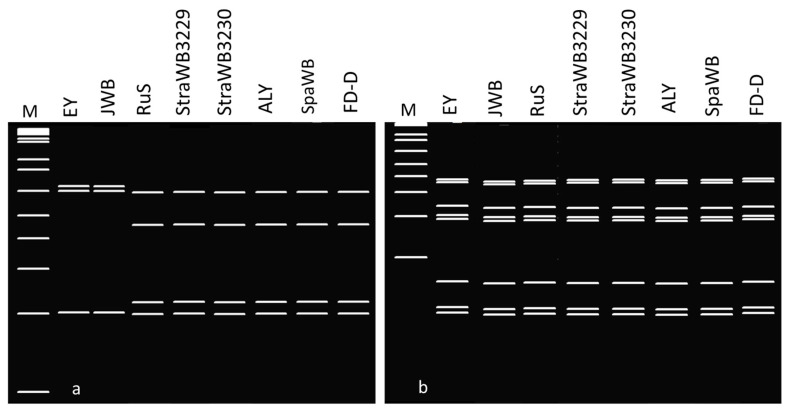
(**a**) *Bfa*I and (**b**) *Tsp*509I virtual restriction profiles of 16S rDNA sequences from strawberry witches’-broom (StraWB) phytoplasma strains detected in southern Italy and other 16SrV group phytoplasmas generated by AcaClone Software, version 1.1.147 (http://www.acaclone.com). M, Invitrogen 1 Kb Plus DNA ladder; EY, elm yellows; JWB, Jujube witches’-broom, RuS, rubus stunt; StraWB3229 and StraWB3230, newly detected StraWB phytoplasma strains (examples); ALY, alder yellows; SpaWB, spartium witches’-broom; FD-D, Flavescence dorée (subgroup 16SrV-D). The 7 bp fragment that is obtained following digestion with *Tsp*509I enzyme of StraWB and RuS phytoplasma strain sequences, which distinguishes these strains from other 16SrV group phytoplasmas, is not evident in the above virtual gel. The virtual gels were labeled and cropped with the software program Photoshop CS3, version 10.0.1 (www.adobe.com). See Table S1 for GenBank accession Nos.

Virtual RFLP analysis of the entire 16S rDNA sequence from StraWB phytoplasma strains with *Mse*I, *Alu*I, *Hae*III, *Tha*I, *Sau*3AI, *Rsa*I, *Hpa*II, *Bfa*I, *Tsp*509I and *Taq*I restriction endonucleases showed uniform restriction profiles with each endonuclease. The *Alu*I, *Hae*III, *Tha*I, *Sau*3AI, *Rsa*I, *Hpa*II, *Bfa*I, *Tsp*509I and *Taq*I restriction profiles were identical to those of all RuS phytoplasma strains examined and resulted from the presence of 5, 1, 6, 5, 7, 3, 3, 9 and 4 restriction sites, respectively. The *Mse*I restriction profiles were identical to those of RuS phytoplasma strain RUS-Ca.P.rubi, RuS400, RuS971, RuSR19, RUS, RS, 28_2018 and 142_2021, which resulted from the presence of 14 restriction sites, but differed from those of strains blackPort and clone three. The latter had an additional *Mse*I site at position 840 and lacked a *Mse*I site at position 610, respectively. Also, StraWB phytoplasma strains had the same *Mse*I, *Alu*I, *Hae*III, *Tha*I, *Sau*3AI and *Taq*I restriction profiles as 16SrV-A, 16SrV-B, 16SrV-C and 16SrV-D subgroup phytoplasmas, the same *Rsa*I and *Hpa*II restriction profiles as 16SrV-B subgroup phytoplasmas and the *Bfa*I restriction profiles as 16SrV-C and 16SrV-D subgroup phytoplasmas. However, StraWB phytoplasma strains were clearly distinguished from other 16SrV group phytoplasmas by the presence of the *Tsp*509I restriction site at position 1151 which is not present in the 16S rDNA sequences of 16SrV-A, 16SrV-B, 16SrV-C and 16SrV-D subgroup phytoplasmas (Figure 8). Therefore, the strawberry-infecting phytoplasma strains occurring in southern Italy, like the RuS agent ‘*Ca*. Phytoplasma rubi’, are members of the 16SrV-E subgroup, which was delineated by Davis and Dally [60].

**Figure 9:**
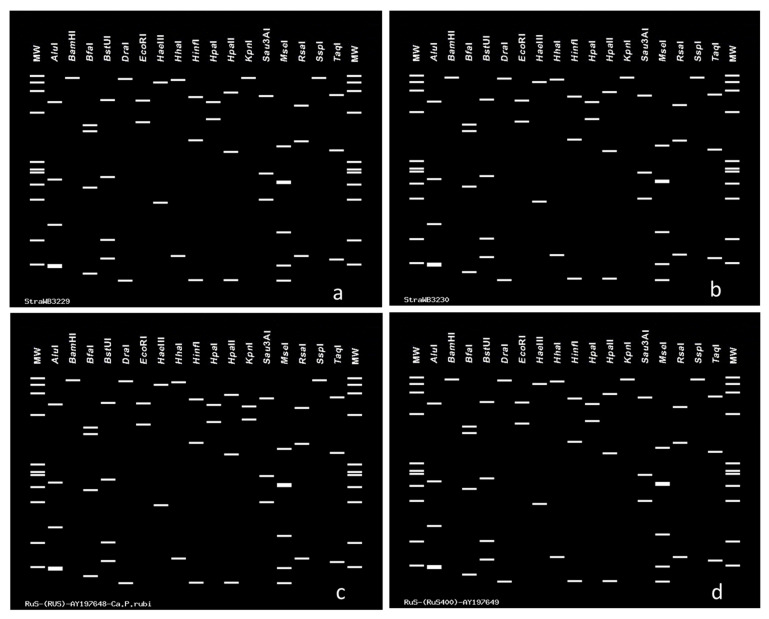
(**a**) Through. (**d**) Examples of virtual RFLP analysis performed through the iPhyClassifier online tool at http://plantpathology.ba.ars.usda.gov/cgi-bin/resource/iphyclassifier.cgi (accessed on 22 July 2025) [61] using 17 restriction enzymes of the 16S rDNA R16F2n/R2 fragments from strawberry witches’-broom (StraWB) phytoplasma strains StraWB3229 (**a**) and StraWB3230 (**b**) and the RuS phytoplasma reference strains RUS (**c**) and RuS400 (**d**). MW, φX174DNA *Hae*III digest. See Table S1 for GenBank accession numbers.

Also, virtual RFLP analysis of the 16S rDNA sequences encompassing the F2nR2 fragment of the StraWB phytoplasma strains using *Alu*I, *Bam*HI, *Bfa*I, *Bst*UI, *Dra*I, EcoRI, *Hae*III, *Hha*I, *Hin*fI, *Hpa*I, *Hpa*II, *Kpn*I, *Mse*I, *Rsa*I, *Sau*3AI, *Ssp*I and *Taq*I restriction endonucleases, showed the same restriction profiles with each enzyme. These profiles, except *Kpn*I, were identical to those of the reference strain RUS of the RuS agent ‘*Ca*. Phytoplasma rubi’. *Kpn*I cleavage sites were not present in the R16F2n/R2 sequences of StraWB phytoplasma strains and RuS phytoplasma strains except strain RUS of the RuS agent ‘*Ca*. Phytoplasma rubi’. The latter had a *Kpn*I restriction site at position 833 (Figure 9).

Following virtual digestion of rp(V)F1A/rp(V)R1A sequences with *Mse*I, *Tsp*509I, *Alu*I, *Taq*I, *Hha*I, *Dra*I and *Ssp*I restriction enzymes, all 14 StraWB phytoplasma strains examined showed the same RFLP profiles with each endonuclease. These profiles were identical to those of RuS phytoplasma strains and resulted from the presence of 29, 17, 10, 1, 1, 8 and 4 restriction sites, respectively. None of the rp(V)F1A/rp(V)R1A sequences showed *Hpa*II and *Hae*III cleavage sites. On the basis of *Mse*I, *Tsp*509I and *Hha*I restriction profiles, StraWB phytoplasma strains were clearly differentiated from other 16SrV group phytoplasmas such as EY1, ALY, FD-C, FD-D, SpaWB229, HD1, PY-In and JWB strains (Table S2). Thus, StraWB phytoplasma strains are members of the ribosomal protein (rp)V-I subgroup delineated by Lee et al. [62]. This subgroup also includes the RuS phytoplasma strains [62].

## 3. Discussion

This study provides evidence on the occurrence of a new phytoplasma disease of cultivated strawberry in the Campania region of southern Italy which is mainly characterized by symptoms resembling those of strawberry witches’-broom and multiplier diseases occurring in North America [3]. In addition, flower and fruit abnormalities, notably virescence, phyllody and elongated sepals were associated in some instances with the newly recorded disease. These symptoms are not reported to be present in strawberry witches’-broom and multiplier diseases [3,45]. Phytoplasma diseases of cultivated strawberry have been reported from several European countries, including Italy [6,20,21,22,27,53,57]. However, there is no published information on the presence in this continent of phytoplasma diseases of strawberry which have a considerable symptom resemblance or are identical symptomatologically to strawberry witches’-broom and multiplier diseases. The only exception is the report by Kronenberg [63], who described a strawberry disorder in the Netherlands similar to the strawberry witches’-broom disease previously described in western Oregon by Zeller [44]. However, the phytoplasma etiology of the strawberry disorder in the Netherlands was never been investigated and confirmed in the following years.

With PCR assays using universal phytoplasma primers and primers specific to the elm yellows (EY) phytoplasma group or 16SrV group, all symptomatic strawberry plants examined in the present study tested phytoplasma positive. However, for a number of diseased plants, visible PCR products were obtained only through nested-PCR assays. This may indicate that the target DNA was present in low concentration or was unevenly distributed in the material examined. Alternatively, PCR inhibitory factors may occur in DNA extracts from plants. Data obtained from sequence, phylogenetic and virtual RFLP analyses of PCR-amplified rDNA, *rpsV* (*rpl22*) and *rpsC* (*rps3*), *map*, *imp* and *groEL* gene sequences, clearly indicated that the diseased strawberry plants harbored phytoplasma strains which were identical or nearly identical to each other and to strains of the rubus stunt (RuS) agent ‘*Ca*. Phytoplasma rubi’, a member of the 16SrV group, subgroup 16SrV-E. The name strawberry witches’-broom (StraWB) phytoplasma strains is proposed for these strawberry-infecting prokaryotes. Because all StraWB phytoplasma strains differed in a few nucleotides in the 16S rDNA sequence from the reference strain RUS of ‘*Ca*. Phytoplasma rubi’, they are regarded as ‘related’ to ‘*Ca*. Phytoplasma rubi’, in accordance with IRPCM’s guidelines [14]. Neither phytoplasmas of the other taxonomic groups nor ‘*Ca*. Phlomobacter fragariae’ could be detected in diseased strawberry plants. Virtual RFLP analysis is a straightforward method which is widely used in phytoplasmology for 16Sr group/subgroup classification [15,16,61]. It also proved highly suitable in our work, in which StraWB phytoplasma strains could be clearly distinguished from other 16SrV group phytoplasmas.

The RuS phytoplasma is an important prokaryotic pathogen that infects wild and cultivated *Rubus* species and hybrids in Europe. In all affected *Rubus* species and cultivars, the symptoms are basically the same, that is, development of numerous, thin, and erect shoots and an excessive lateral branching of the whole plant, with phyllody and proliferation of flowers [64,65,66,67]. This organism is closely related to the elm yellows (EY), alder yellows (ALY), spartium witches’-broom (SpaWB) agents as well as the phytoplasmas causing Flavescence dorée (FD) of grapevine and forms with them, together with a few other phytoplasmas, a distinct taxonomic group, the EY phytoplasma group or 16SrV group. The members of this group are only distantly related to other phytoplasmas [59,62]. With exception of the strain blackPort, which was identified in diseased blackberry (*Rubus* sp.) plants in Portugal and was assigned to the 16SrV-I subgroup [68], all RuS phytoplasma strains which were molecularly characterized are members of the 16SrV-E subgroup [60,62]. Our data shows that the StraWB phytoplasma strains are members of the same 16SrV-E subgroup. The RuS phytoplasma strains seem to have a narrow host range. They preferentially infect plants only in the genus *Rubus*. However, they were also detected by PCR assays in a few plants of great mallow (*Malva sylvestris*) showing proliferation symptoms and of wild dog rose (*Rosa canina*) which were non-symptomatic, growing in the proximity of European stone fruit yellows-infested orchards in France [69]. The RuS phytoplasma was transmitted from naturally diseased *Rubus* plants to the experimental host *Catharanthus roseus* (periwinkle) via dodder (*Cuscuta* spp.) bridges [70]. *Apium graveolens*, *Chrysanthemum carinatum*, *Trifolium repens*, *Fragaria vesca* and strawberry plants are also reported as experimental hosts which were inoculated through insect vectors [71,72]. However, in these inoculated test plants, the identity of the infecting phytoplasma has never been determined with molecular methods. Experimentally infected plants of *Fragaria vesca* and cultivated strawberry showed symptoms of witches’-broom, severe growth reduction and phyllody [71]. The RuS agent is naturally transmitted by the *Rubus* leafhoppers of the genus *Macropsis*, including *M. fuscula* [73,74,75]. These leafhoppers live monophagously on *Rubus* plants. In transmission experiments, the froghopper *Philaenus spumarius* and the leafhoppers *Allygus mayri* and *Euscelis plebeja* were able to transmit the pathogen [76,77]. It seems unlikely that *P. spumarius*, *A. mayri* and *E. plebeja* play an important role in the natural spread of the disease between *Rubus* species because they do not live specifically on *Rubus* plants. However, they may be able to transmit the disease agent occasionally between *Rubus* and other plant species due to their polyphagous character. Although *M. fuscula* is not able to develop on strawberry, it transmitted the RuS agent from raspberryto strawberryin laboratory experiments [71]. Infection of RuS agents in strawberry, however, has never been observed in the field, although in some cases strawberry and RuS-affected raspberry plants usually were grown side by side on the same farm. Thus, to our best knowledge, this is the first report on the occurrence of RuS phytoplasma in naturally affected strawberry plants. As mentioned above, most of the symptoms observed on affected strawberry plants in southern Italy are similar to those of strawberry witches’-broom and multiplier diseases in North America. However, the similarity of symptoms observed does not allow for the conclusion that the diseases occurring in other countries are caused by the RuS agent. It is well known in phytoplasmology that similar symptoms in a given plant host may be induced by taxonomically different phytoplasmas [11,13].

The present study expands knowledge on the natural host range of RuS phytoplasma and confirms and further extends previous work showing that the RuS agent is a homogeneous pathogen throughout Europe [59]. All strains of this taxon originating from different geographical areas and plant hosts, including the newly detected StraWB strains, proved to be identical or nearly identical on the basis of the genes examined. Prior to this work, the relationships of the RuS agent with closely related phytoplasmas at 16S/23S rDNA spacer region, *map*, *imp* and *groEL* gene sequence levels, were based only on sequence analysis of a few strains or were largely unknown. Thus, the phylogenetic relationships of RuS and StraWB phytoplasma strains in the mentioned genes were extensively examined for the first time in the present work. In southern Italy, 16SrI group phytoplasmas were previously detected in strawberry plants with symptoms of flower virescence and fruit phyllody and in some, potential insect vectors [78,79,80]. Evidence of the presence of these phytoplasmas was not obtained in the present study.

RuS-affected wild blackberry (*R. fruticosus*) plants have been observed in southern Italy, including the area where the investigated strawberry disease occurred [81,82]. It is likely that these plants act as an initial source of inoculum from which transmission to strawberry plants occurred through occasional strawberry-feeding leafhoppers leading to StraWB disease. However, cross-inoculation experiments need to be carried out in order to prove definitively that authentic disease agents have been transmitted. On the other hand, StraWB phytoplasma strains may have been introduced to southern Italy through infected strawberry planting stock originating from foreign nurseries. Further work is needed to investigate epidemiological aspects of the newly recorded strawberry disease, including insect vectors and role of alternative hosts. Information on these aspects is essential for appropriate control practices.

## 4. Materials and Methods

### 4.1. Sampling, DNA Isolation, Primers and PCR Amplification

Symptomatic and non-symptomatic strawberry plants were sampled from March 2024 to June 2024 in the three above-mentioned commercial fields, which belong to the same fruit-growing farmer. The plants were grown under open plastic tunnels in organic cropping systems and consisted of the cultivar Marimbella. A total of 60 diseased strawberry plants were sampled, 20 plants in each field. The plants were repeatedly inspected to monitor symptom development and disease progression. Healthy strawberry plants maintained in insect-proof screenhouses were used as healthy controls. DNA samples of a number of previously characterized phytoplasma strains including rubus stunt (RuS), elm yellows (EY), alder yellows (ALY), spartium witches’-broom (SpaWB), Flavescence dorée of grapevine (FD), stolbur (STOL) of pepper, American aster yellows (AAY) and Western X-disease [83,84], extracted from the experimental host periwinkle or the respective original hosts, were used for comparisons.

For DNA isolation, approximately 1.0 g of fresh tissue which consisted mainly of leaf petioles, midribs, branch crowns and flower parts taken from symptomatic and non-symptomatic strawberry plants, was used. DNA was extracted according to the phytoplasma enrichment procedure previously reported by Ahrens and Seemüller [85]. PCR amplification was carried out using both ribosomal and non-ribosomal DNA (rDNA) primers, as outlined in Table S3. The following universal phytoplasma primer pairs were employed: P1/P7, P1A/P7A and R16F2n/R2, which amplify rDNA fragments of approximately 1850 bp, 1820 bp and 1240 bp in length, respectively [62,86,87]. Group-specific phytoplasma primer pairs employed, directed to rDNA sequences, were as follows: fB1/rULWS that specifically amplifies a fragment of about 1650 bp from the phytoplasmas of the elm yellows group or 16SrV group [88], fStol/rStol which specifically amplifies a fragment of about 620 bp in length from the phytoplasmas of the stolbur group or 16SrXII group [89], fAY/rAY that specifically amplifies a 324 bp fragment from phytoplasmas of the aster yellows group or 16SrI group [90] and R16(III)F2/R1 that specifically amplifies a fragment of about 800 bp from phytoplasmas of the X-disease group or 16SrIII group [91]. In nested PCR assays, the amplification products obtained with universal primer pair P1/P7 were re-amplified with either universal primer pairs P1A/P7A and R16F2n/R2 or with group-specific primer pairs fB1/rULWS, fStol/rStol, fAY/rAY and R16(III)F2/R1. For amplification of non-rDNA sequences, the following EY group-specific primer pairs were used: rp(V)F1/rpR1 followed by rp(V)F1A/rp(V)R1A which amplifies a DNA fragment of about 1200 bp in length of ribosomal protein (rp) genes *rpsV* (*rpl22*) and *rpsC* (*rps3*) [62], FD9f5/MAPr1 followed by FD9f6/MAPr2 which amplifies a 674 bp fragment of SecY-map locus (*map* gene) [92], fEY_imp/rpyrG that amplifies a fragment of 675 bp of *imp* gene [93] and fEY_groEL/rEY_groEL that amplifies a fragment of 880 bp of *groEL* gene [93]. In addition to phytoplasma primers, the primer pair Fra4/Fra5 which amplifies a 550 bp 16S rDNA fragment from ‘*Ca*. Phlomobacter fragariae’ [56], was also used.

Amplifications were performed with the Applied Biosystems VeritiPro 96-Well Thermal Cycler (Thermo Fisher Scientific, Waltham, MA, USA) in a 50 µL reaction containing 125 µM of the four dNTPs, 0.5 µM of each primer, 1U of Dream Taq DNA polymerase, 1x polymerase buffer (both Thermo Fisher Scientific), 5 µL of template DNA (100–200 ng) and water. For the second round of amplification with nested primers, either 1 μL of undiluted PCR products or 3 μL of 1:40 dilution obtained in the initial amplification were used as template in the same reaction mixture as in the first round. PCR conditions including annealing temperature, extension time and number of cycles for each primer pair were as previously described [56,84,87,90,91]. Also, agarose gel electrophoresis of the PCR products was carried out as described [84].

### 4.2. Sequencing, Phylogenetic and Virtual RFLP Analyses

Amplicons obtained with universal phytoplasma primer pairs P1/P7 and P1A/P7A and group-specific primer pairs rp(V)F1A/rp(V)R1A, FD9f6/MAPr2, fEY_imp/rpyrG and fEY_groEL/rEY_groEL from fourteen StraWB phytoplasma strains, were purified through the PureLink PCR Purification Kit (Invitrogen, Waltham, MA, USA) and sequenced directly by the commercial sequencing facilities of BMR Genomics, University of Padua, Padua, Italy. Amplicons for sequencing were obtained from a representative number of positive samples which were collected from different parts of the fields surveyed and from plants with different disease severity. Sequencing of both strands of each amplicon was accomplished using the same primers employed in PCR amplification. In addition, for the sequencing of P1/P7 and P1A/P7A amplicons, the following internal primers were used: fU5, R16F2n, rU3 and R16R2 [87,94]. Sequencing data were assembled and edited using either DNASTAR’s LaserGene software (DNASTAR), version 7.1 or the sequence analysis software BioEdit, version 7.2.5 [95]. Consensus sequences were used in a BLAST, version 2.17.0 search analysis carried out online at https://blast.ncbi.nlm.nih.gov/Blast.cgi [96]. Multiple sequence alignments were performed using CLUSTAL W of the LaserGene software (DNASTAR) or of the BioEdit software (BioEdit). Sequence similarities were calculated through the MEGALIGN program of the LaserGene software (DNASTAR). Sequences generated in the present research were deposited in the GenBank database under the following accession numbers: PX122035 to PX122048 for rDNA, PX121979 to PX121992 for *rpsV* (*rpl22*) and *rpsC* (*rps3*), PX121993 to PX122006 for *map*, PX122021 to PX122034 for *imp* and PX122007 to PX122020 for *groEL* genes.

Phylogenetic analysis was performed through the genetic analysis software MEGA, version 12 using the neighbor-joining parameters and the bootstrap test [58]. The data were resampled 1000 times and the bootstrap percentage values are given at the nodes of the tree. Neighbor-joining and maximum likelihood methods yielded similar results in tree construction. Phylogenetic distances were calculated by pairwise comparison.

Virtual RFLP analyses of rDNA and rp sequences using key restriction enzymes for the differentiation of 16SrV group phytoplasmas and, thus, for the determination of 16Sr group and subgroup affiliations, were performed through the pDRAW32 program developed by AcaClone Software (http://www.acaclone.com) and the iPhyClassifier online tool at http://plantpathology.ba.ars.usda.gov/cgi-bin/resource/iphyclassifier.cgi (accessed on 22 July 2025) and [61,62,97]. For GenBank accession numbers of sequences compared in this study and rp subgroup designation see Tables S1 and S2 [98,99,100,101,102,103,104,105,106,107,108,109,110].

## 5. Conclusions

The new witches’-broom disease of cultivated strawberry observed in southern Italy is symptomatologically similar to strawberry witches’-broom and multiplier diseases occurring in North America, except for flower and fruit abnormalities in some instances. The newly observed strawberry disease proved to be associated with phytoplasma strains, the strawberry witches’-broom (StraWB) phytoplasma strains, which were identical or nearly identical to each other and to strains of the rubus stunt (RuS) agent ‘*Ca*. Phytoplasma rubi’, a member of the 16SrV group, subgroup 16SrV-E. This pathogen was previously not known to occur in naturally infected strawberry plants. Since RuS-affected wild blackberry plants are present in the area where the investigated strawberry disease occurred, it is likely that these plants act as pathogen reservoirs from which transmission to strawberry plants occurred through occasional strawberry-feeding leafhoppers. It is also possible that StraWB phytoplasma strains were introduced to southern Italy through infected strawberry planting stock originating from foreign nurseries. The present study confirms and further extends previous work showing that the RuS agent is a homogeneous pathogen throughout Europe. All strains of this taxon originating from different geographical areas and plant hosts, including the newly detected StraWB strains, proved to be identical or nearly identical on the basis of genes so far examined. Further work is required to study the vector–phytoplasma–plant relationships of the newly recorded strawberry disease.

## Data Availability

DNA sequences generated in the present research were deposited in the GenBank database under the following accession numbers: PX122035 to PX122048 for rDNA, PX121979 to PX121992 for *rpsV* (*rpl22*) and *rpsC* (*rps3*), PX121993 to PX122006 for *map*, PX122021 to PX122034 for *imp*, and PX122007 to PX122020 for *groEL* genes.

## Supplementary Materials

The following supporting information can be downloaded at: https://www.mdpi.com/article/10.3390/plants14182914/s1, Figure S1: Diseased cultivated strawberry plants associated with strawberry witches’-broom (StraWB) phytoplasma strain infections; Figure S2: Strawberry witches’-broom (StraWB)-affected cultivated strawberry plants; Figure S3: Phylogenetic tree constructed using the neighbor-joining method with *map* gene sequences from strawberry witches’-broom (StraWB) phytoplasma strains detected in southern Italy; Table S1: Phytoplasmas related to strawberry witches’-broom (StraWB) phytoplasma strains examined in this study; Table S2: Summary of the pattern types (subgroups) produced by virtual RFLP analysis of ribosomal protein (rp) gene sequences [rp(V) F1A/rp(V)R1A fragments] of the strawberry witches’-broom (StraWB) phytoplasma strains; Table S3: Details of oligonucleotide primers used in this study.
