# Supplementary material for: A Witches’-Broom Disease of Cultivated Strawberry Associated with ‘Candidatus Phytoplasma Rubi’-Related Strains in Southern Italy
Source: Plants (Basel). 2025 Sep 19;14(18):2914. doi: 10.3390/plants14182914 (PMC12473155; doi:10.3390/plants14182914)
Supplement: Supplementary file 1 [file plants-14-02914-s001.zip › Supplementary Figures-Straw.pdf]

Supplementary Figures

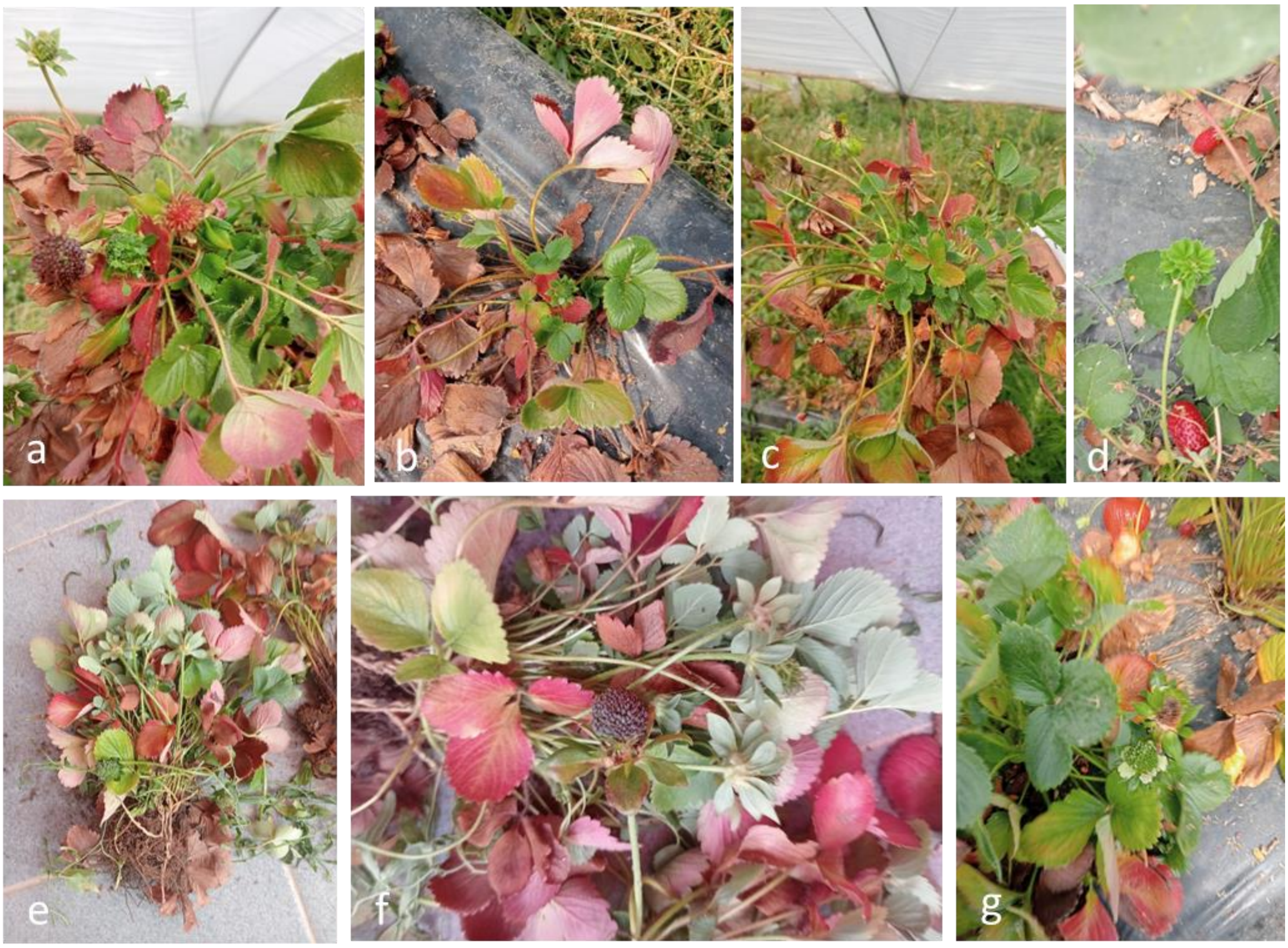

Figure S1. Diseased cultivated strawberry plants associated with strawberry witches'-broom (StraWB) phytoplasma strain infections. Symptoms of crown proliferation (**a**, **c**, **e**, and **f**), reddening (**a** through **c**, **e** and **f),** virescent petals with phyllody (**a**, **b**, **c**, and **g**), elongated sepals (**e** and **f**), aborted berries (**a** and **f**), entirely phylloid flower (**d**).

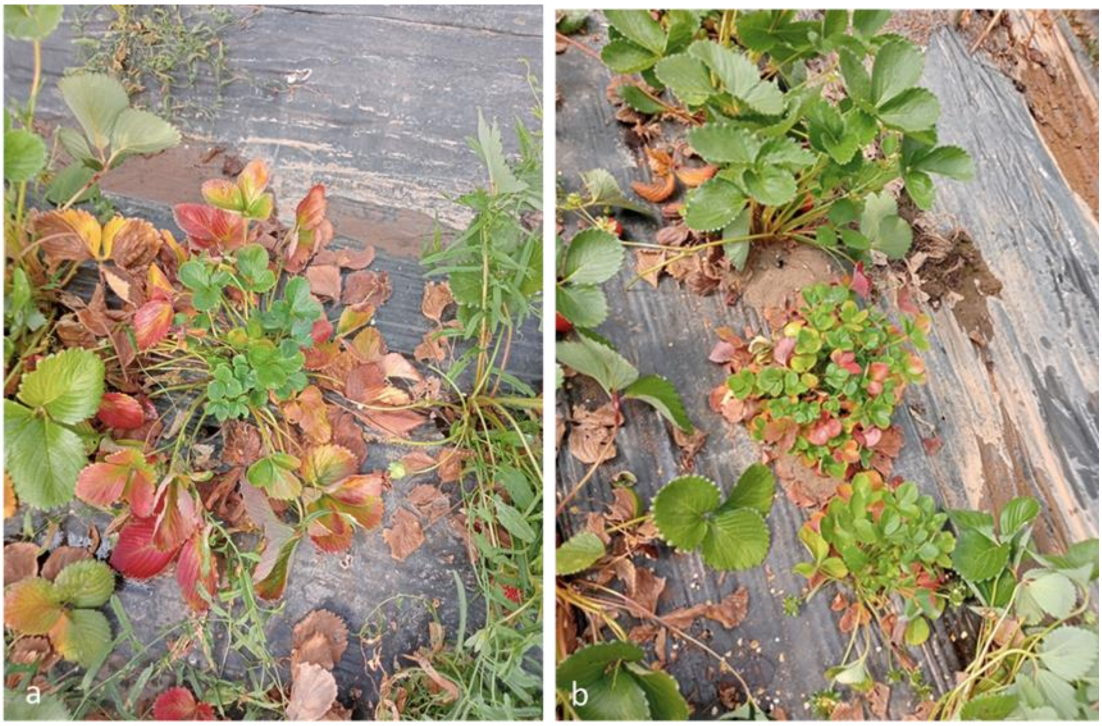

Figure S2. Strawberry witches'-broom (StraWB)-affected cultivated strawberry plants. (**a and b**) Young leaves show yellowing symptoms and are generally cupped upwardly whereas older leaves often redden, lay flat on the ground, and soon turn brown. (**b**) Two adjacent diseased plants in the border row of the plastic tunnels among healthy-appearing strawberry plants.

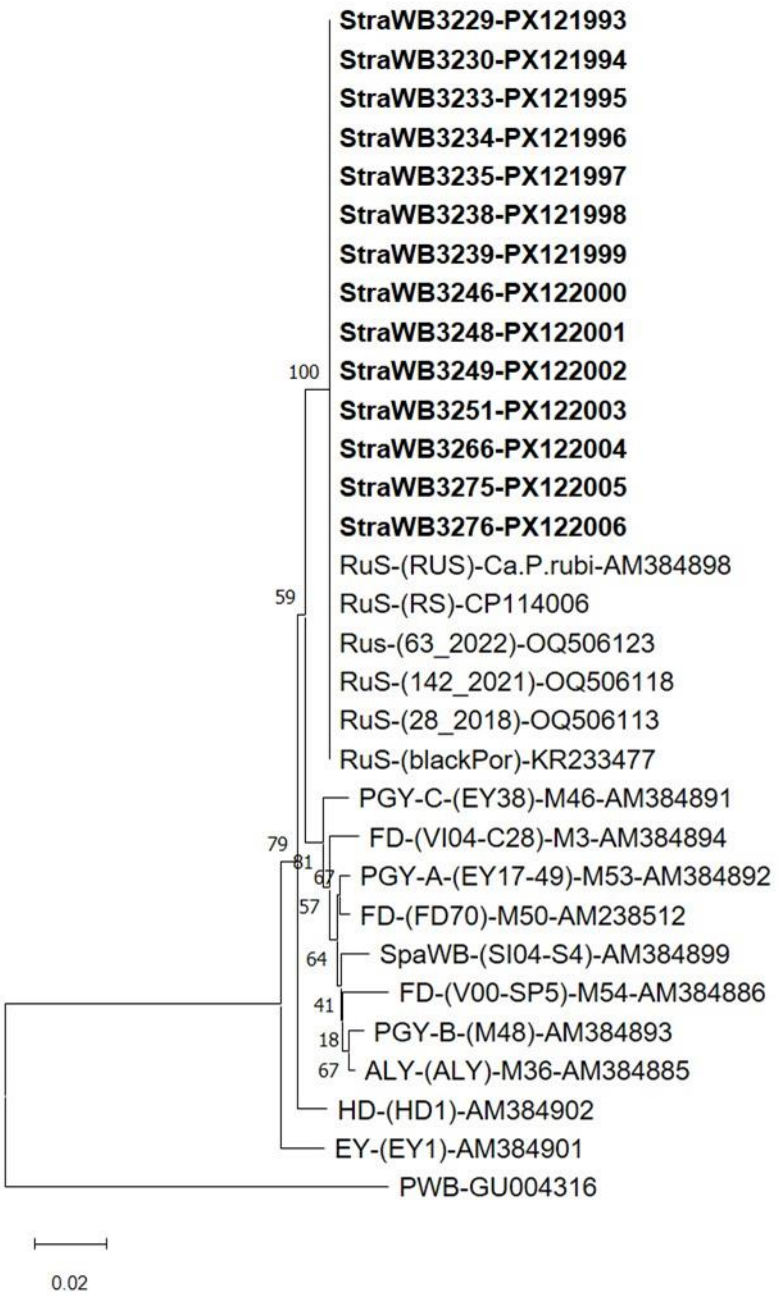

Figure S3. Phylogenetic tree constructed using the neighbor-joining method [58] with *map* gene sequences from strawberry witches'-broom (StraWB) phytoplasma strains detected in southern Italy (in bold type), rubus stunt (RUS), Palatinate grapevine yellows (PGY), flavescence dorée (FD), spartium witches'-broom (SpaWB), alder yellows (ALY), hemp dogbane yellows (HD) and elm yellows (EY) phytoplasma strains. Potato witches'-broom (PWB) phytoplasma strain PWB was used as the outgroup. Bar represents a phylogenetic distance of 0.02 nucleotide substitutions per site. GenBank accession number is provided for each phytoplasma. Bootstrap values are shown on branches of the phylogenetic tree.
