# Supplementary material for: A Witches’-Broom Disease of Cultivated Strawberry Associated with ‘Candidatus Phytoplasma Rubi’-Related Strains in Southern Italy
Source: Plants (Basel). 2025 Sep 19;14(18):2914. doi: 10.3390/plants14182914 (PMC12473155; doi:10.3390/plants14182914)
Supplement: Supplementary file 1 [file plants-14-02914-s001.zip › Supplementary Tables-Straw.pdf]

Supplementary Tables

Table S1. Phytoplasmas related to strawberry witches'-broom (StraWB) phytoplasma strains examined in this study.

Phytoplasma (strain)	Original host	Geographical origin	Sequence*	GenBank accession No.	Reference/collector(s)
Rubus stunt (RUS)-Ca. P. rubi	*Rubus fruticosus*	Italy	16S	AY197648	[59, 62]
Rubus stunt (RuS400)	*Rubus fruticosus*	Italy	16S	AY197649	[62]
Rubus stunt (RuS971)	*Rubus idaeus*	Switzerland	16S	AY197650	[62]
Rubus stunt (RuSR19)	*Rubus caesius*	Germany	16S	AY197651	[62]
Rubus Stunt (RUS)	*Rubus fruticosus*	Italy	16S, SR	Y16395	[98]
Rubus stunt (RS)	*Rubus caesius*	Germany	16S, SR	CP114006	[99]
Rubus stunt (28_2018)	*Rubus idaeus*	Czech Republic	16S, SR	OQ520100	[67]
Rubus stunt (142-2021)	*Rubus idaeus*	Czech Republic	16S, SR	OQ520101	[67]
Rubus stunt (clone 3)	*Rubus plicatus*	Czech Republic	16S, SR	OQ520102	[67]
Rubus stunt (blackPort)	*Rubus* spp.	Portugal	16S, SR	KR233473	[68]
Elm yellows (EY1)-Ca. P. ulmi	*Ulmus americana*	USA	16S	AY197655	[62]
Elm yellows (EY1)	*Ulmus americana*	USA	16S, SR	AF122910	[100]
Alder yellows (ALY)	*Alnus glutinosa*	Italy	16S	AY197646	[62]
Alder yellows (ALY-SI)	*Alnus glutinosa*	Italy	16S, SR	Y16387	[98]
Spartium witches'-broom (SpaWB229)	*Spartium junceum*	Italy	16S	AY197652	[62]
Hemp dogbane yellows (HD1)	*Apocynum cannabinum*	USA	16S	AY197654	[62]
Virginia creeper (VC)	*Parthenocissus quinquefolia*	USA	16S, SR	AF305198	[62]
Flavescence dorée (FD-C)	*Vitis vinifera*	Italy	16S	AY197645	[62]
Flavescence dorée (FD-D)	*Vitis vinifera*	Italy	16S	AY197644	[62]
Flavescence dorée (FD70)	*Vitis vinifera*	France	16S, SR	AF176319	[60]
Flavescence dorée (CH)	*Vitis vinifera*	Switzerland	16S, SR	CP097583	[101]
Jujube witches'-broom (JWB-G1)-Ca. P. ziziphi	*Ziziphus jujuba*	China	16S	AB052876	[102]
Balanites witches'-broom (BltWB)-Ca. P. balanitae	*Balanites triflora*	Myanmar	16S, SR	AB689678	[103]
Japanese raisin witches'-broom (JRWB)	*Hovenia dulcis*	South Korea	16S, SR	AB442218	[104]
Peach yellows (PY-In)	*Prunus persica*	India	16S	AY197660	[62]
Jujube witches'-broom (JWB)	*Ziziphus jujuba*	China	16S	AY1976661	[62]
Rubus stunt (RUS)	*Rubus fruticosus*	Italy	*rpsV* (*rpl22*), *rpsC* (*rps3*)	AY197668	[62]
Rubus stunt (RuS400)	*Rubus fruticosus*	Italy	*rpsV* (*rpl22*), *rpsC* (*rps3*)	AY197669	[62]
Rubus stunt (RuS971)	*Rubus idaeus*	Switzerland	*rpsV* (*rpl22*), *rpsC* (*rps3*)	AY197670	[62]
Rubus stunt (RuSR19)	*Rubus caesius*	Germany	*rpsV* (*rpl22*), *rpsC* (*rps3*)	AY197671	[62]
Rubus stunt (RS)	*Rubus caesius*	Germany	*rpsV* (*rpl22*), *rpsC* (*rps3*)	CP114006	[99]
Rubus stunt (28-2018)	*Rubus idaeus*	Czech Republic	*rpsV* (*rpl22*), *rpsC* (*rps3*)	OQ506116	[67]
Rubus stunt (63_2022)	*Rubus fruticosus*	Czech Republic	*rpsV* (*rpl22*), *rpsC* (*rps3*)	OQ506126	[67]
Rubus stunt (blackPort)	*Rubus* spp.	Portugal	*rpsV* (*rpl22*), *rpsC* (*rps3*)	KR233475	[68]
Rubus stunt (RuS-L)	*Rubus idaeus*	Lithuania	*rpsV* (*rpl22*), *rpsC* (*rps3*)	HM104661	[Ivanauskas A., Valiunas D., Davis R.E.]
Elm yellows (EY1)	*Ulmus americana*	USA	*rpsV* (*rpl22*), *rpsC* (*rps3*)	AY197675	[62]
Alder yellows (ALY)	*Alnus glutinosa*	Italy	*rpsV* (*rpl22*), *rpsC* (*rps3*)	AY197666	[62]
Flavescence dorée (FD-D)	*Vitis vinifera*	Italy	*rpsV* (*rpl22*), *rpsC* (*rps3*)	AY197664	[62]
Flavescence dorée (FD-C)	*Vitis vinifera*	Italy	*rpsV* (*rpl22*), *rpsC* (*rps3*)	AY197665	[62]
Spartium witches'-broom (SpaWB229)	*Spartium junceum*	Italy	*rpsV* (*rpl22*), *rpsC* (*rps3*)	AY197672	[62]
Hemp dogbane yellows (HD1)	*Apocynum cannabinum*	USA	*rpsV* (*rpl22*), *rpsC* (*rps3*)	AY197674	[62]
Peach yellows (PY-In)	*Prunus persica*	India	*rpsV* (*rpl22*), *rpsC* (*rps3*)	AY197680	[62]
Jujube witches'-broom (JWB)	*Ziziphus jujuba*	China	*rpsV* (*rpl22*), *rpsC* (*rps3*)	AY197681	[62]
Rubus stunt (RUS)-Ca. P. rubi	*Rubus fruticosus*	Italy	*map*	AM384898	[92]
Rubus stunt (RS)	*Rubus caesius*	Germany	*map*	CP114006	[99]
Rubus stunt (63-2022)	*Rubus fruticosus*	Czech Republic	*map*	OQ506123	[67]
Rubus stunt (142_2021)	*Rubus idaeus*	Czech Republic	*map*	OQ506118	[67]
Rubus stunt (28-2018)	*Rubus idaeus*	Czech Republic	*map*	OQ506113	[67]
Rubus stunt (blackPort)	*Rubus* spp.	Portugal	*map*	KR233477	[68]
Elm yellows (E04-D714)	*Ulmus glabra*	France	*map*	AM384901	[92]
Palatinate grapevine yellows-PGY-A-(EY17-49)-M53	*Vitis vinifera*	Germany	*map*	AM384892	[92]
Palatinate grapevine yellows-PGY-B-(M48)-M48	*Vitis vinifera*	Germany	*map*	AM384893	[92]
Palatinate grapevine yellows PGY-C-(EY38)-M46	*Vitis vinifera*	Germany	*map*	AM384891	[92]
Alder yellows (ALY)-M36	*Alnus glutinosa*	Italy	*map*	AM384885	[92]

Spartium witches'-broom (SI04-S4)	*Spartium junceum*	Italy	*map*	AM384899	[92]
Hemp dogbane yellows (HD1)	*Apocynum cannabinum*	USA	*map*	AM384902	[92]
Flavescence dorée (FD70)-M50	*Vitis vinifera*	France	*map*	AM238512	[92]
Flavescence dorée (V00-SP5)-M54	*Vitis vinifera*	France	*map*	AM384886	[92]
Flavescence dorée (VI04-C28)-M3	*Vitis vinifera*	Italy	*map*	AM384894	[92]
Rubus stunt (RS)	*Rubus caesius*	Germany	*imp*	CP114006	[99]
Elm yellows (ULW)	*Ulmus minor*	France	*imp*	MT418908	[93]
Elm yellows (EY2470)	*Ulmus* sp.	Italy	*imp*	PP332858	[105]
Flavescence dorée (CH)	*Vitis vinifera*	Switzerland	*imp*	CP097583	[101]
Flavescence dorée (FD-D)	*Vitis vinifera*	Italy	*imp*	MK614707	[106]
Flavescence dorée (FD-C-Piemonte)	*Vitis vinifera*	Italy	*imp*	KJ402359	[107]
Flavescence dorée (FD70)	*Vitis vinifera*	France	*imp*	MT668500	[93]
Jujube witches'-broom (Jwb-nky)	*Ziziphus jujuba*	China	*imp*	MG818479	[Gao R., Wang J., Lu X.]
Rubus stunt (RS)	*Rubus caesius*	Germany	*groEL*	CP114006	[99]
Elm yellows (ULW)	*Ulmus minor*	France	*groEL*	MT418907	[93]
Elm yellows (1986-Um-RP)	*Ulmus minor*	Germany	*groEL*	MT638084	[93]
Alder yellows (ALY)	*Alnus glutinosa*	Italy	*groEL*	MT638097	[93]
Alder yellows (ALY2923)	*Alnus* sp.	Italy	*groEL*	PP230411	[84]
Flavescence dorée (CH)	*Vitis vinifera*	Switzerland	*groEL*	CP097583	[101]
Flavescence dorée (FD70)	*Vitis vinifera*	France	*groEL*	MT638098	[93]
Jujube witches'-broom (Jwb-nky)	*Ziziphus jujuba*	China	*groEL*	CP025121	[108]
Jujube witches'-broom (Hebei-2018)	*Ziziphus jujuba*	China	*groEL*	CP091835	[109]

* 16S, 16S rDNA; SR, 16S/23S rDNA spacer region

Table S2. Summary of the pattern types (subgroups) produced by virtual RFLP analysis of ribosomal protein (rp) gene sequences [rp(V) F1A/rp(V)R1A fragments] of the strawberry witches'-broom (StraWB) phytoplasma strains detected in southern Italy and rp sequences retrieved from the GenBank database. Numbers shown in each column represent distinct RFLP types with each enzyme.

Strain	Genbank Accession. No.	rpV subgroup*	RFLP pattern types								
			*Hpa*II	*Dra*I	*Taq*I	*Alu*I	*Mse*I	*Ssp*I	*Tsp509*I	*Hae*III	*Hha*I
RUS**	AY197668	I	1	3	1	1	8	1	9	1	4
RuS400**	AY197669	I	1	3	1	1	8	1	9	1	4
RuS971**	AY197670	I	1	3	1	1	8	1	9	1	4
RuSR19**	AY197671	I	1	3	1	1	8	1	9	1	4
RS**	CP114006	I	1	3	1	1	8	1	9	1	4
28_2018**	OQ506116	I	1	3	1	1	8	1	9	1	4
63_2022**	OQ506126	I	1	3	1	1	8	1	9	1	4
blackPort**	KR233475	I	1	3	1	1	8	1	9	1	4
RuS-L**	HM104661	I	1	3	1	1	8	1	9	1	4
EY1**	AY197675	A	1	1	1	1	1	1	1	1	1
ALY-(ALY)**	AY197666	H	2	3	1	1	3	2	4	1	1
FD-(FD-C)**	AY197665	D	1	3	1	1	4	1	5	1	1
FD-(FD-D)**	AY197664	E	2	3	4	1	7	1	5	1	1
SpaWB-(SpaWB229)**	AY197672	L	2	4	5	1	3	3	8	1	1
HD1**	AY197674	J	1	3	4	1	5	1	6	1	1
PY-In**	AY197680	B	1	3	2	2	2	1	2	2	3
JWB**	AY197681	C	1	3	3	3	2	1	3	3	3
StraWB3229	PX121979	I	1	3	1	1	8	1	9	1	4
StraWB3230	PX121980	I	1	3	1	1	8	1	9	1	4
StraWB3233	PX121981	I	1	3	1	1	8	1	9	1	4
StraWB3234	PX121982	I	1	3	1	1	8	1	9	1	4
StraWB3235	PX121983	I	1	3	1	1	8	1	9	1	4
StraWB3238	PX121984	I	1	3	1	1	8	1	9	1	4
StraWB3239	PX121985	I	1	3	1	1	8	1	9	1	4
StraWB3246	PX121986	I	1	3	1	1	8	1	9	1	4
StraWB3248	PX121987	I	1	3	1	1	8	1	9	1	4
StraWB3249	PX121988	I	1	3	1	1	8	1	9	1	4
StraWB3251	PX121989	I	1	3	1	1	8	1	9	1	4
StraWB3266	PX121990	I	1	3	1	1	8	1	9	1	4
StraWB3275	PX121991	I	1	3	1	1	8	1	9	1	4
StraWB3276	PX121992	I	1	3	1	1	8	1	9	1	4

* rp subgroup designation according to Lee et al. [62] and Martini et al. [110].

** Sequences retrieved from the GenBank database.

Table S3. Details of oligonucleotide primers used in this study.

Primer	Primer sequence (5'-3')	Sense	Target	References
P1	AGA-GTT-TGA-TCC-TGG-CTC-AGG-A	Forward	16S rDNA	[86]
P7	CGT-CCT-TCA-TCG-GCT-CTT	Reverse	23S rDNA	[86]
P1A	AAC-GCT-GGC-GGC-GCG-CCT-AAT-AC	Forward	16S rDNA	[62]
P7A	CCT-TCA-TCG-GCT-CTT-AGT-GC	Reverse	23S rDNA	[62]
R16F2n	GAA-ACG-ACT-GCT-AAG-ACT-GG'	Forward	16S rDNA	[87]
R16R2	TGA-CGG-GCG-GTG-TGT-ACA-AAC-CCC-G	Reverse	16S rDNA	[87]
fB1	GAC-CCT-TCA-AAA-GGT-CTT-AG	Forward	16S rDNA	[88]
rULWS	GTC-TTT-TAT-ATA-AGA-GAA-ACA-C	Reverse	16S/23S rDNA spacer region	[88]
fStol	GCC-ATC-ATT-AAG-TTG-GGG-A	Forward	16S rDNA	[89]
rStol	AGA-TGT-GAC-CTA-TTT-TGG-TGG	Reverse	16S/23S rDNA spacer region	[89]
fAY	GCA-CGT-AAT-GGT-GGG-CAC-TT	Forward	16S rDNA	[90]
rAY	CGA-AGT-TAG-GCC-ACC-GGC-TTT	Reverse	16S rDNA	[90]
R16(III)F2	AAG-AGT-GGA-AAA-ACT-CCC	Forward	16S rDNA	[91]
R16(III)R1	TTC-GAA-CTG-AGA-TTG-A	Reverse	16S rDNA	[91]
rp(V)F1	TCG-CGG-TCA-TGC-AAA-AGG-CG	Forward	*rpsS*	[62]
rp(V)R1	ACG-ATA-TTT-AGT-TCT-TTT-TGG	Reverse	*rplP*	[62]
rp(V)F1A	AGG-CGA-TAA-AAA-AGT-TTC-AAA-A	Forward	*rpsS*	[62]
rp(V)R1A	GGC-ATT-AAC-ATA-ATA-TAT-TAT-G	Reverse	*rplP*	[62]
FD9f5	CAA-AAA-ATT-ACT-TTT-GGC-GGG-AC	Forward	*SecY*	[92]
MAPr1	TGC-TCA-AAA-TGA-GCG-CTT-AAA-C	Reverse	*map*	[92]
FD9f6	GTC-GCT-TTA-GAA-TCG-ACA-CA	Forward	*SecY*	[92]
MAPr2	TCG-GAA-GTA-ACA-GCA-GTC-CA	Reverse	*map*	[92]
fEY_imp	CAT-TTT-AAA-TAC-TGT-ATA-TTA-AAT-AC	Forward	*imp*	[93]
rpyrG	GAC-CTT-TTA-AAC-CAC-ATC-C	Reverse	*imp*	[93]
fEY_groEL	GTT-AAT-GAT-GGC-GTT-ACA-ATC-GC	Forward	*groEL*	[93]
rEY_groEL	GTT-AAA-GAA-GGA-CTT-TTA-TCC-GC	Reverse	*groEL*	[93]
Fra4	CTC-CTC-TGT-CTC-TAA-AAG	Forward	16S rDNA	[56]
Fra5	AGC-AAT-TGA-CAT-TAG-CGA'	Reverse	16S rDNA	[56]
